# A comprehensive database of thermal developmental plasticity in reptiles

**DOI:** 10.1038/sdata.2018.138

**Published:** 2018-07-17

**Authors:** Daniel W. A. Noble, Vaughn Stenhouse, Julia L. Riley, Daniel A. Warner, Geoffrey M. While, Wei-Guo Du, Tobias Uller, Lisa E. Schwanz

**Affiliations:** 1 Evolution & Ecology Research Centre, School of Biological, Earth, and Environmental Sciences, University of New South Wales, Sydney, New South Wales 2052, Australia; 2 School of Biological Sciences, Victoria University, Wellington 6037, New Zealand; 3 Department of Biological Sciences, Macquarie University, Sydney, New South Wales 2109, Australia; 4 Department of Biological Sciences, Auburn University, Auburn, Alabama 36849, USA; 5 School of Biological Sciences, University of Tasmania, Hobart, TAS 7005, Australia; 6 Institute of Zoology, Chinese Academy of Sciences, Beijing 100101, China; 7 Department of Biology, Lund University, Sölvegatan 37, 223 62 Lund, Sweden

**Keywords:** Ecophysiology, Evolutionary developmental biology, Herpetology, Phenology

## Abstract

How temperature influences development has direct relevance to ascertaining the impact of climate change on natural populations. Reptiles have served as empirical models for understanding how the environment experienced by embryos can influence phenotypic variation, including sex ratio, phenology and survival. Such an understanding has important implications for basic eco-evolutionary theory and conservation efforts worldwide. While there is a burgeoning empirical literature of experimental manipulations of embryonic thermal environments, addressing widespread patterns at a comparative level has been hampered by the lack of accessible data in a format that is amendable to updates as new studies emerge. Here, we describe a database with nearly 10, 000 phenotypic estimates from 155 species of reptile, collected from 300 studies manipulating incubation temperature (published between 1974–2016). The data encompass various morphological, physiological, behavioural and performance traits along with growth rates, developmental timing, sex ratio and survival (e.g., hatching success). This resource will serve as an important data repository for addressing overarching questions about thermal plasticity of reptile embryos.

## Background & Summary

Conditions experienced early in life are known to impact phenotypes in profound ways that can have long-lasting effects on fitness^1,2^. Understanding the extent to which developmental environments impact phenotypes is important for addressing many fundamental questions in ecology and evolutionary biology^3–5^, as well as predicting the effect environmental change will have on populations both locally and globally^1,6^. Ectothermic vertebrates in particular are sensitive to variation in early developmental temperature, which often is mediated by local climatic conditions, landscape features as well as maternal nest site choice or maternal basing behaviour. In reptiles (turtles and tortoises, tuatara, lizards, snakes and crocodilians), there is a growing empirical literature testing the effects of early thermal environments (i.e., incubation temperatures) on the phenotypic development of a broad range of physiological, morphological and performance traits^7–11^. Nonetheless, there is currently no database collating and summarising this vast literature in a way that is amenable to updates as the literature grows or that can be expanded to address not only questions on the impacts of temperature, but also other environmental conditions (e.g., moisture, pH) that may be relevant to phenotypic development and survival.

Here, we describe a large database on the effects of incubation temperature on phenotypic traits in oviparous reptiles. Our database differs from others (e.g., BioTraits^12^), in that it focuses primarily on thermal developmental plasticity by collating studies manipulating temperatures experienced during pre-hatching developmental periods only. Furthermore, ours is the first database on thermal developmental plasticity to provide an updatable platform summarising phenotypic effects of incubation temperatures. A smaller, preliminary version, of the dataset was thoroughly analysed in a related manuscript^1^ and future plans are to expand the data to capture other environmental drivers of phenotypic variation, such as moisture, pH, and oxygen concentrations. In addition, although the database focuses on oviparous species, it can be expanded to include environmental effects on embryos of viviparous species.

As the database grows, we believe that it can be used to address a wide variety of questions. For example, some of the questions that are currently or have previously been addressed with the database include:

- *What are the overall magnitude of effects of incubation duration on phenotypic development?* Qualitative syntheses of this research area have provided an unclear picture of both the magnitude of effect temperature has on phenotypic development, and whether complex patterns alluded to in these reviews^13–15^ can be explained by species-specific or study-level attributes. Using aspects of these data Noble *et al.*^1^ have shown strong overall effects, independent of temperature differences between studies, but found little support for the hypothesis that much of the variation in effects could be explained by phylogeny. Nonetheless, more robust phylogenetic analyses may provide greater insight.
- *Do the effects of early thermal conditions persist late in life?* The life-long consequences of adverse early environmental conditions are topics of both theoretical and applied interest, and while immediate effects are well documented^2,16^, we still know little about whether long-term effects exist, and if so, what the consequences are for population dynamics and life-history evolution^16^. Noble *et al.*’s^1^ analysis suggests that thermal environments can affect the phenotype later in life, however, a more detailed analysis of what predicts variation among species will be worthwhile.
- *Do extreme developmental temperatures elicit developmental stress?* Novel environmental conditions, including extreme temperatures, are predicted to affect phenotypic variation^17^ and can lead to compromised survival^1^ through developmental stress. We are currently using the database on a more targeted set of traits explore the generality of this prediction.
- *What are the shapes of developmental reaction norms?* While previous analyses suggest that many thermal reaction norms for traits exhibit the expected ‘thermal performance curve’ shape^1^, we are currently exploring the database in more detail on sub-samples of the data to understand the shapes of reaction norms for specific traits that are highly represented in the data (e.g., body size and mass).
- *How realistic thermal fluctuations change the impact thermal developmental environments have on phenotypic development?* Previous analyses suggest that more natural, fluctuating conditions decrease the magnitude of phenotypic effects. However, as the database grows and more detailed and realistic thermal conditions are applied experimentally, resolving uncertainty surrounding this question will be possible. It will also be important in establishing the impact thermal conditions have in nature, where temperatures typically vary on daily and longer time scales.
- *How does thermal developmental plasticity facilitate or impede invasion success and adaptation to changing climatic conditions?* Phenotypic plasticity is predicted to play an important role in early stages of adaptation to changing environmental conditions^17,18^ or novel environments (as encountered by invasive species^19^) and thermal plasticity is expected to feature strongly in this process for ectotherms. As the database grows and thermal reaction norms are more thoroughly characterized in more species these questions maybe feasibly addressed.

The Reptile Development Database can be accessed freely online via a user-friendly webpage (www.repdevo.com) that stores and lodges all versions of past databases in addition to the most up-to-date version. This ensures reproducibility of analyses as the database is updated and evolves to include new types of data. Unpublished data can be submitted through downloaded data templates, and queries can be sought by emailing the team (contact details are on the webpage).

## Methods

We searched for published literature (1974–2016) describing experiments that manipulated incubation temperature (i.e., pre-hatching developmental period only) in reptiles in *Web of Science* (v5.13.2) using the following ‘title’ or ‘abstract’ search terms: temperature* AND incubat*, along with one of the following: reptil*, lizard*, squamat*, snake*, turtle*, chelon*, testudin*, crocodil*, alligator*, tuatara*, sphenodon*. In addition, we considered all citations in three major reviews of the topic^13–15^, and included any additional papers from these sources not identified in our searches. For data currently included in this database, the studies had the following attributes: 1) research on an oviparous reptile (Class Reptilia; excluding birds); 2) employ an experimental manipulation of incubation temperature of eggs; 3) present data on hatching success, incubation duration, or post-hatching phenotypes; 4) consist of eggs that did not receive exogenous hormone application and yolk removal; 6) there was not a substantial delay between oviposition and experimental incubation (e.g., >48-hours). In some cases, papers could not be accessed and/or were in languages that were non-translatable. While we attempted to translate where possible, those that could not be were excluded from the database (approximately 8 studies). We included 684 publications from the primary scientific literature that were relevant based on the title of the paper. If exclusion of papers was not possible based on the title of the publication, we assigned a unique identification number to each publication and considered the abstract and full text for exclusion/inclusion criteria. ‘Citations.csv’ (Table 1; [Data Citation 1]) details the publications considered based on their full-text, and we provide reasons for their exclusion (if excluded) along with full reference information.

**Figure 1:**
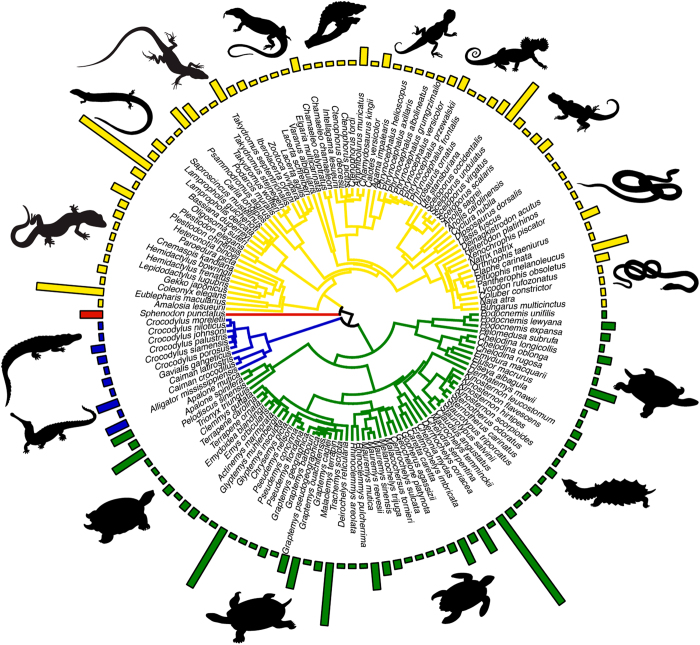
Phylogenetic tree of 140 taxa present within the database. Tree was derived from TimeTree.org ^21^. Each of the four major orders are represented in the database (‘blue’ – Crocodilia; ‘red’ – Tuatara; ‘yellow’ – Squamates; and ‘green’ – Testudines). Bars above taxa indicate the number of studies (scaled by a factor of 10) for each species. Note that 15 taxa were excluded because of ambiguity surrounding their taxonomic position.

**Figure 2:**
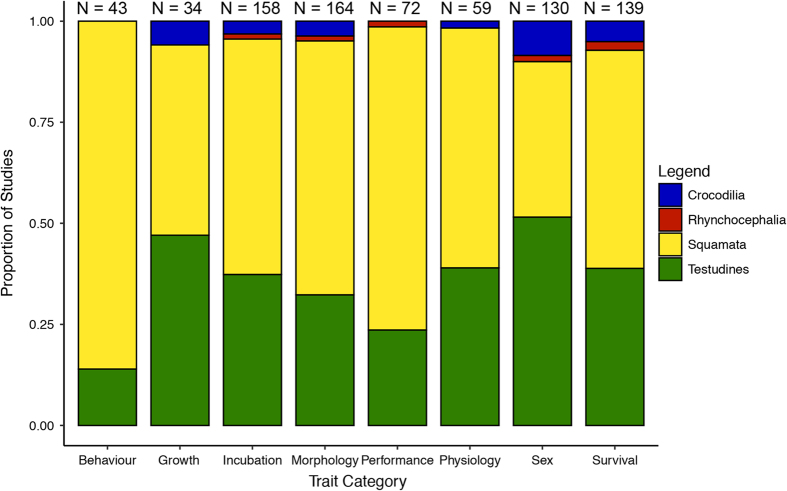
Proportion of studies containing each of the general trait categories (Behaviour, Growth, Incubation, Morphology, Performance, Physiology, Sex and Survival), grouped by the specific taxonomic order. For definitions of the specific trait types included in each of these categories refer to Table 2 (available online only). N above the bars are the number of studies.

Following exclusion / inclusion based on the criteria we had a dataset of 300 publications from which we extracted complete or partial data. From each publication, we extracted data into ‘Database.csv’ as outlined in Table 2 (available online only) [Data Citation 1]. This included the focal information of incubation temperature regime, phenotypes of study, and summary results [mean, error, sample size – type of error (e.g., standard error, standard deviation or 95% confidence interval) is indicated using a separate column – see Table 2 (available online only)] for each phenotype and incubation treatment. We also noted the age of the specimens (assumes to be age 0, hatchlings, if not indicated) in the sample, the temperature fluctuation of treatments, the sex of the sample (assumes to be mixed if not indicated), whether the data were raw or adjusted (e.g., least square means) phenotypic means and the location of the population sample. Data were taken from text or tables from each manuscript, however, when data were provided in figures we extracted key information from these figures (assuming they were clear and readable) using DataThief^20^. In addition, we recorded contextual information regarding species, study design, cofactors included, and comments. Taxonomic naming was standardised using TimeTree.org^21^. Genus and species names not identified (N=15 species) in TimeTree were cross checked with the EMBL Reptile Database^22^. Missing data were coded as ‘NA’. All traits measured were classified into one of eight Trait Categories (see Table 2, (available online only)). Taxonomic representation of data was highest in the Orders Squamata (lizards and snakes – N=75 species) and Testudines (turtles and tortoises – N=69 species) in terms of raw numbers of studies (Fig. 1), although representation relative to species richness was highest in Orders Rhynchocephalia (tuatara, 1 species) and Crocodilia (alligators and crocodiles, – N=10 species). Traits classified under the category morphology were the most-commonly collected data (Fig. 2).

These methods, as well as further exclusion criteria, were used for and described in a meta-analysis of a subset of the data^1^.

### Code Availability

Code for technical validation (see below) can be found on the Zenodo archived repository [Data Citation 1].

## Data Records

### Data Record 1

RepDevo is hosted by GitHub (https://github.com/RepDevo/ReptileDevelopmentDatabase) and provided a unique, stable DOI on Zenodo [Data Citation 1]. This zip file contains csv files of the: 1) citation information, 2) extracted data and 3) metadata.

## Technical Validation

We have implemented a number of tests that check the database prior to new releases and major updates by using the *testthat*^23^ package in R. The tests check the database for structural integrity (i.e., its internal organization), variable consistency (i.e., correct naming rules) as well as data integrity checks (i.e., outliers, correct data types). Additionally, sub-samples of the data have been thoroughly checked by multiple collectors prior to the release of v1.0.0. The online database can also easily be expanded and corrected if errors are identified. Data will be updated annually with new studies and any errors identified corrected.

## Usage Notes

Regularly-updated versions of the Reptile Development Database (RepDevo) can be found at, and downloaded from: www.repdevo.com. The Database.csv, Citations.csv and Metadata.csv files are located on external servers hosted by GitHub and are fully version controlled. Releases will be lodged on GitHub and version releases will be provided with a unique, stable DOI and permanently stored for improved reproducibility as new updates and errors are documented. This is achieved using Zenodo’s DOI and versioning capabilities (https://doi.org/10.5281/zenodo.1188482). Users can cite within the manuscript the specific version used along with its DOI if necessary, which will be provided with the version downloaded from the webpage (www.repdevo.com). However, minimally the specific version should be specified when the data are used to ensure reproducibility of any resulting analyses.

We have been fairly inclusive in our database and many studies report or manipulate a multitude of factors at once, including moisture conditions, oxygen concentrations or measure phenotypes at different ages, temperatures or post-incubation treatments. Our database contains all these data, and so, any future work should take care to extract relevant data based on the question of interest. We have been careful to indicate the population, species, moisture conditions and any post hatching manipulations (e.g., temperature) or measurements (e.g., age) that data are derived from. Some studies confound incubation treatment with year and we have tried to be careful in identifying the specific year in which the experimental manipulation took place to ensure that comparisons of temperatures across different years is not undertaken. In addition, experimental designs can vary substantially among studies (e.g., random allocation of eggs to treatment, split-clutch designs, etc.). This variation could influence results and care should be taken when calculating effect sizes and comparing results of studies with different designs. To account for this, we have also categorized experimental designs that can be considered by users.

## Additional information

**How to cite this article**: Noble, D. W. A. *et al*. A comprehensive database of thermal developmental plasticity in reptiles. *Sci. Data* 5:180138 doi: 10.1038/sdata.2018.138 (2018).

**Publisher’s note**: Springer Nature remains neutral with regard to jurisdictional claims in published maps and institutional affiliations.

## Supplementary Material



## Figures and Tables

**Table 1:** Database of papers examined at the abstract or full-text level for extractable phenotypic data.

Column	Description
*paper_no*	Unique number assigned to each manuscript data was extracted from. This *paper_no* column matches the *data_no* column in Database.csv.
*first_author_surname*	The surname of the first author on the manuscript data was extracted from
*authors*	All authors on the manuscript, and ordered as specified when published
*year*	Year of manuscript publication
*title*	Title of the publication
*journal*	The name of the journal the article was published in
*volume*	Volume of the journal the publication belongs to
*pages*	The range of pages of the publication
*doi_stableurl*	An internet link to the publication as appropriate. Typically, this is either its DOI, a stable URL, or a link to the publication on ResearchGate. If unable to locate a URL, then cell filled with “NA”
*abstract*	Abstract of the publication
*used*	“Y”: the publication was included in the database
	“N”: the database was excluded from the database a “Partially”: a subset of the data within the paper was included in the database
*excld_1*	Excluded (Y/N) because the study species was viviparous
*excld_2*	Excluded (Y/N) because the methods or data were unclear
*excld_3*	Excluded (Y/N) because the study involved a designed temperature shift experiment
*excld_4*	Excluded (Y/N) because the study was not an experimental manipulation
*excld_5*	Excluded (Y/N) because the study involved hormonal experimental manipulation
*excld_6*	Excluded (Y/N) because post-hatching treatments were confounded with the temperature treatments
*excld_7*	Excluded (Y/N) because the paper was inaccessible
*excld_8*	Excluded (Y/N) for another reason, that is not specified above. More details can be found in the 'comments' column as appropriate.
*comments*	Text to specify comments for any other reason the paper was excluded, or to provide more details about the manuscript.
Full citation details and exclusion criteria are included in “Citations.csv” file.	

**Table 2:** Database of study descriptors and data extracted from published studies.

Column	Values	Description
*data_no*	Integer from 1000 onwards	Unique number assigned to each manuscript or unpublished dataset . This *data_no* column matches the *paper_no* column in Citations.csv.
*first_author_surname*	Variable	The surname of the first author on the manuscript data was extracted from
*pub_year*	Variable	Year the manuscript data was extracted from was published
*order*	Crocodilia	Order of the reptile of study species
	Rhynchocephalia	
	Squamata	
	Testudines	
*family*	Variable	Family of the reptile of the study species
*genus*	Variable	The genus name of the study species
*species*	Variable	The species name of the study species
*genus_timetree*	Variable	The genus name of the study species in the time-tree database (http://www.timetree.org/). When a study species was not found genus name was kept the same as the paper and cross checked with the EMBL Reptile Database..
*species_timetree*	Variable	The species name of the study species in the time-tree database (http://www.timetree.org/). When a study species was not found species name was kept the same as the paper and cross checked with the EMBL Reptile Database.
*latin_name*	Variable	The full latin name (genus and species) of the reptile of study
*specific_location_name*	String of location descriptions	Specific location of the study as specified within the manuscript. If more than one detail is provided a comma separates them. Also, if the study animal were sourced from a captive colony or hatchery this is specified in this column. If location data is not specified in the manuscript, these columns are filled in with an ‘NA’.
*city*	Variable	The city in which the study was conducted as specified within the manuscript. If this is not specified in the manuscript, these columns are filled in with an ‘NA’.
*state_province*	Variable	The state or province in which the study was conducted as specified within the manuscript. If this is not specified in the manuscript, these columns are filled in with an ‘NA’.
*country*	Variable	The country in which the study was conducted as specified within the manuscript. If this is not specified in the manuscript, these columns are filled in with an ‘NA’.
*population*	Variable	“pop1”, “pop2”, etc: if means within a manuscript were from independent populations (e.g., different species within the same location, across different geographic locations)
*data_year*	Mostly numeric	If the year data was collected was specified within the manuscript it is detailed here in a numeric format (i.e., 2018). If data was from multiple years, these are listed and separated by a comma in a string (i.e., 2016, 2017, 2018).
		If study methods indicate that data were collected within a particular year, but the numeric date was not specified, then we have used the test “year” with a number after it (i.e., “year1”). If data were collected over multiple years (pooled), but no numeric dates specified, then these are listed in a string using this alphanumeric format (i.e., “year 1, year2”).
		If study year is unspecified, this is coded as “NA”.
*egg_design*	independent	Reflecting how eggs were allocated to each incubation treatment.
	independent_pseudo	“independent”: each incubation treatment consisted of eggs from independent females with no repeated samples from a single female clutch
	other	“independent_pseudo”: each incubation treatment treated eggs from the same female as replicates, but there were separate clutches within each treatment (i.e. eggs were not split across treatments)
	split_clutch	“split_clutch”: distributed a single egg from each clutch within each of the incubation treatments
	split_psuedo	“split_psuedo”: distributed eggs from a single clutch evenly across multiple incubation treatments, but eggs from the same clutch were in the same incubation treatment
		“other”: not possible to determine the egg allocation strategy
*source_page*	Numeric	The page within the manuscript that the data was extracted from
*source*	Variable FigNo.	The specific location within the manuscript from where the data was extracted; or ‘author’ if data were supplied via contact with an author of the publication
	Variable TableNo.	
	Text	
	Author	
*trait_cat*	Incubation	Each trait was assigned to one of seven traits categories. Examples of traits included in each category include: Incubation – time to hatching in days; Behaviour – Activity, latency to a behaviour; Growth – growth rates of various body regions (snout-vent length, mass, head width and length); Morphology – Mass, snout-vent-length, head width and length, carapace width and length, usually presented in mm and grams; Performance – Sprint speed, maximal run distance, time to run, swimming speed, bite force and pull force, usually in m/s, m, Newtons (N); Physiology – generally hormone concentrations (e.g. testosterone, estrogen), metabolic rate (e.g., VC02/ V02 per time), Carcass ash, dry mass, energy and water content; Survival – primarily contains hatching success, but also survival to a particular age.
	Growth	
	Morphology	
	Performance	
	Physiology	
	Survival	
*trait*	Variable	The detailed name of the phenotypic trait that relates to the specific name used within the original source.
*simp_trait*	Variable	A simplified version of the name of the phenotypic traits that was used in the manuscript in order to produce summary figures for publication. This column is likely to be of most use to users given that it provides a simplified indication of the trait quantified within the study.
*units*	Variable	The units that were used to quantify a phenotypic trait
*egg_embryo_*	Egg	Specification of the life stage at which the phenotypic trait was measured.
*hatchling*	Embryo	“Egg”: measurements of the egg or portions of the egg prior to the embryo hatching
	Hatchling	“Embryo”: measurements of pre-hatched embryos. Often embryos were removed from eggs prior to hatching and measured on a given trait.
		“Hatchling”: measurements of offspring at- or post-hatching.
*age*	Numeric	The age (days post-hatching) at which the phenotypic trait was measured. For survival, the age is the second census date. Egg and embryo measurements given NA.
*sex*	females	“females”: all females in sample
	males	“males”: all males in sample
	mixed	“mixed” mixture of sexes in the sample, or if the sample was unknown
*other_factors*		Describes if the phenotypic trait means integrated other confounding factors that were not otherwise specified in the database (e.g., post-hatching treatments, genetic strains or races, elevation, etc.); otherwise “NA”
*const_fluct*	Const	Simplified categorization of incubation temperature design that is based on the *T_fluc* column. This column can help users distinguish different experimental designs (see definitions below) across studies. “Range” is defined as the full range of temperatures. For example, if 23 +/− 0.5C, then the range is 1 C
	Fluct	“Const”: range of daily temperature data ≤1 deg. C
	Irreg	“Fluct”: range of daily temperature data was ≥ 1 deg. C
	Shift	“Irreg”: temperature profiles vary idiosyncratically, or there was a slight delay between oviposition and the establishment of incubation treatments.
		“Shift”; temperature profiles shifted during incubation between established temperature treatments as part of the experiment design
*T*	Numeric	Mean temperature, in degrees Celcius, of the incubation treatment
*T_fluc*	Numeric	The range of temperature, in degrees Celcius, of the incubation treatment. For example, if the temperature varied between 20 C and 30 C, with a mean temperature of 25 C this column would contain a 10 (+/− 5 C around the mean). This column can be used to treat temperature fluctuations on a continuous scale or classify experimental designs (as is done in *const_fluct*) in a user specific way.
*water_potential*	Numeric	Water potential of treatment, if specified. Negative integers within this column represent water potential measured in kPa. The other representations of water potential reflect a wetter/drier treatment using the terms used in the manuscript. If water potential was not specified within the paper, or if it was specified using a water to vermiculite ratio, then the uncertainty in specific water potential value was represented using “NA”.
*mean*	Numeric	The mean of the phenotypic trait from the incubation treatment
*error*	Numeric	The error of the phenotypic trait from the incubation treatment
*N*	Numeric	The sample size of the phenotypic trait from the incubation treatment
*error_type*	SE	The type of error associated with the data
	SD	“SE”: standard error
	CI	“SD”: standard deviation
	P	“CI”: 95% confidence intervals
	NA	“P”: the data was a proportion of individuals within the treatment, and thus did not have error
		“NA”: If the error type was not unspecified
*data_style*	adj	Specification if the data were either raw, or adjusted in some way (e.g., residuals, log transformation, etc.).
	raw	
*comment_code*	Numeric	Numbers associated with a comment code to specify particular issues associated about the papers. These were listed in ascending order, separated by a comma followed by a space. The comment codes represent:
		1=other
		2=we assumed or calculated N
		3=N reported within the paper is conflicting
		4=calculated error from paper
		5=assumed error type
		6=error uncertain or missing
		7=unclear temperature treatment
		8=back-calculated hatching success from provided sample sizes
		9=data pooled between two+ factors because of presentation or non-significant effects
		10=N is missing
		11=calculated age
		12=assumed trait units through logical deduction
		13=potential confounding factors in experimental design
		14=unknown trait units
		15=citation was used to get experimental information (sex ratio, etc.)
		16=Substantial delay between oviposition and entering treatment
*comm_text*	Text	Explanations, if needed, for the comments codes.
Some data were provided by the study authors. Column descriptors are those found in “Database.csv”.		
